# Suppressing NF-κB and NKRF Pathways by Induced Pluripotent Stem Cell Therapy in Mice with Ventilator-Induced Lung Injury

**DOI:** 10.1371/journal.pone.0066760

**Published:** 2013-06-26

**Authors:** Yung-Yang Liu, Li-Fu Li, Cheng-Ta Yang, Kai-Hsi Lu, Chung-Chi Huang, Kuo-Chin Kao, Shih-Hwa Chiou

**Affiliations:** 1 Chest Department, Taipei Veterans General Hospital, Taipei, Taiwan; 2 Institute of Clinical Medicine, School of Medicine, National Yang-Ming University, Taipei, Taiwan; 3 Department of Medicine, Division of Pulmonary and Critical Care Medicine, Chang Gung Memorial Hospital, Taoyuan, Taiwan; 4 Department of Medicine, Chang Gung University, Taoyuan, Taiwan; 5 Department of Respiratory Therapy, Chang Gung Memorial Hospital, Taoyuan, Taiwan; 6 Graduate Institute of Basic Medicine, Fu Jen Catholic University, New Taipei City, Taiwan; 7 Department of Medical Research and Education, Cheng-Hsin General Hospital, Taipei, Taiwan; 8 Department of Ophthalmology, Taipei Veterans General Hospital, Taipei, Taiwan; 9 Department of Medical Research and Education, Taipei Veterans General Hospital, Taipei, Taiwan; 10 Institute of Pharmacology, School of Medicine, National Yang-Ming University, Taipei, Taiwan; University of Kansas Medical Center, United States of America

## Abstract

**Background:**

High-tidal-volume mechanical ventilation used in patients with acute lung injury (ALI) can induce the release of inflammatory cytokines, as macrophage inflammatory protein-2 (MIP-2), recruitment of neutrophils, and disruption of alveolar epithelial and endothelial barriers. Induced pluripotent stem cells (iPSCs) have been shown to improve ALI in mice, but the mechanisms regulating the interactions between mechanical ventilation and iPSCs are not fully elucidated. Nuclear factor kappa B (NF-κB) and NF-κB repressing factor (NKRF) have been proposed to modulate the neutrophil activation involved in ALI. Thus, we hypothesized intravenous injection of iPSCs or iPSC-derived conditioned medium (iPSC-CM) would decrease high-tidal-volume ventilation-induced neutrophil infiltration, oxidative stress, and MIP-2 production through NF-κB/NKRF pathways.

**Methods:**

Male C57BL/6 mice, aged between 6 and 8 weeks, weighing between 20 and 25 g, were exposed to high-tidal-volume (30 ml/kg) mechanical ventilation with room air for 1 to 4 h after 5×10^7^ cells/kg mouse iPSCs or iPSC-CM administration. Nonventilated mice were used as control groups.

**Results:**

High-tidal-volume mechanical ventilation induced the increases of integration of iPSCs into the injured lungs of mice, microvascular permeability, neutrophil infiltration, malondialdehyde, MIP-2 production, and NF-κB and NKRF activation. Lung injury indices including inflammation, lung edema, ultrastructure pathologic changes and functional gas exchange impairment induced by mechanical ventilation were attenuated with administration of iPSCs or iPSC-CM, which was mimicked by pharmacological inhibition of NF-κB activity with SN50 or NKRF expression with NKRF short interfering RNA.

**Conclusions:**

Our data suggest that iPSC-based therapy attenuates high-tidal-volume mechanical ventilation-induced lung injury, at least partly, through inhibition of NF-κB/NKRF pathways. Notably, the conditioned medium of iPSCs revealed beneficial effects equal to those of iPSCs.

## Introduction

Acute lung injury (ALI) and its most severe manifestation, acute respiratory distress syndrome (ARDS) are marked by increased microvascular permeability and capillary leakage because of severe epithelial and endothelial injury [1], [2]. Pathologic lung over-distension may occur in the healthy parts of the lungs in patients with ARDS employing ventilator support. Mechanical ventilation with high tidal volumes (V_T_) causes ventilator-induced lung injury (VILI) characterized by noncardiogenic pulmonary edema, release of cytokines and chemokines leading to influx of neutrophils [3]. The recruitment of inflammatory cells with high V_T_ ventilation is initiated by enhanced production of inflammatory mediators, such as murine macrophage inflammatory protein-2 (MIP-2), which is a functional homolog of human interleukin-8 (IL-8) in rodents. MIP-2, a potent chemokine for neutrophil, has been known to contribute to the pathogenesis of VILI through recruiting these leukocytes into the lung [4]–[5]. Targeting MIP-2 is therefore of potential therapeutic advantage in the ALI.

Nuclear factor-κB (NF-κB) plays a pivotal role in the pathogenesis of immune and inflammatory responses [6]–[7]. The activation of NF-κB may lead to the expression of MIP-2, tumor necrosis factor-α (TNF-α), and interleukin-1β (IL-1β), which activate inflammatory cascades in the ALI [8]–[9]. We previously demonstrated that high V_T_ ventilation caused a time-dependent increase on NF-κB activation in a mouse VILI model [10]. NF-κB plays an important role in the inflammatory signal transduction elicited in the release of IL-8 by high stretch ventilation *in vitro* and *in vivo* [11]–[12]. Held et al. also showed overventilation triggered activation of NF-κB and elicits release of chemokines and cytokines from perfused lungs similar to that of LPS in mice [13]. Nevertheless, the activity of NF-κB is controlled at further levels including inducible phosphorylation, binding of coactivators, and repressors [14].

NF-κB-repressing factor (NKRF) is a transcriptional factor silencer protein that specifically counteracts the basal activity of several NF-κB-dependent promoters of interleukin-8, interferon β (IFN-β), and inducible nitric oxide synthase (iNOS) genes by direct binding to specific DNA sequences [15]–[16]. NKRF mRNAs are constitutively expressed in all tested human tissues. Endogenous NKRF binds to NF-κB proteins by a direct protein-protein interaction, and is implicated in the inhibition of NF-κB basal activity [17]. Intriguingly, NKRF has been shown to possess a dual role of regulating IL-8 transcription [18]–[19]. NKRF binds to negative regulatory elements (NREs) of the IL-8 promoter and directly interacts with NF-κB to represses the IL-8 gene expression in the basal (un-stimulated) state, but conversely turns to coactivate with NF-κB to induce IL-8 transcription under IL-1 stimulation.

Recent studies demonstrated that induced pluripotent stem cells (iPSCs) could be generated from mouse embryonic fibroblasts (MEFs) as well as from adult human fibroblasts through the retrovirus-mediated transfection of four transcription factors, Oct3/4, Sox2, c-Myc, and Klf4 [20]–[22]. The morphology, proliferative abilities, surface antigens, gene expression, epigenetic status of pluripotent cell-specific genes, and telomerase activity between embryonic stem cells (ESCs) and iPSCs were similar [21]–[22]. In addition to the features of self-renewal and differentiation into three germ layers, iPSCs can be derived from the patient's somatic cells and avoid the ethical controversy and the possibility of immune rejection after transplantation raised in ESCs [23]. Therefore, iPSCs are regarded to be a good candidate for cell therapy and used for autologous transplantation without the risk of rejection. An *in vivo* study of cerebral ischemic rats showed that iPSCs present the capability of multi-lineage differentiation and further reduce the severity of brain infarcts [24]. Our recent study of lipopolysaccharide (LPS)-induced ALI in mice demonstrated that iPSC therapy has anti-inflammatory effects [25]. Of interest, Curley et al. revealed that mesenchymal stem cell (MSC) therapy enhanced lung repair following VILI through a keratinocyte growth factor-dependent paracrine mechanism [26]. However, the roles of iPSC therapy in VILI have not been fully delineated and require further exploration.

In this high mechanical stretch-induced ALI model in mice, we examined the relationships between high V_T_ ventilation, iPSCs and iPSC-derived conditioned medium (iPSC-CM) production of MIP-2, intracellular oxidative stress, and activation of NF-κB and NKRF signaling using pharmacological inhibition with SN-50, a specific inhibitor for NF-κB and short interfering RNA (siRNA) targeted to NKRF. We hypothesized that intravenous injections of either iPSCs or iPSC-CM would decrease neutrophil infiltration, oxidative stress, lung edema, and MIP-2 production in mice exposed to high V_T_ ventilation through modulating NF-κB and NKRF pathways.

## Results

### Characterization of MEF-derived iPSCs

**Figure 1:**
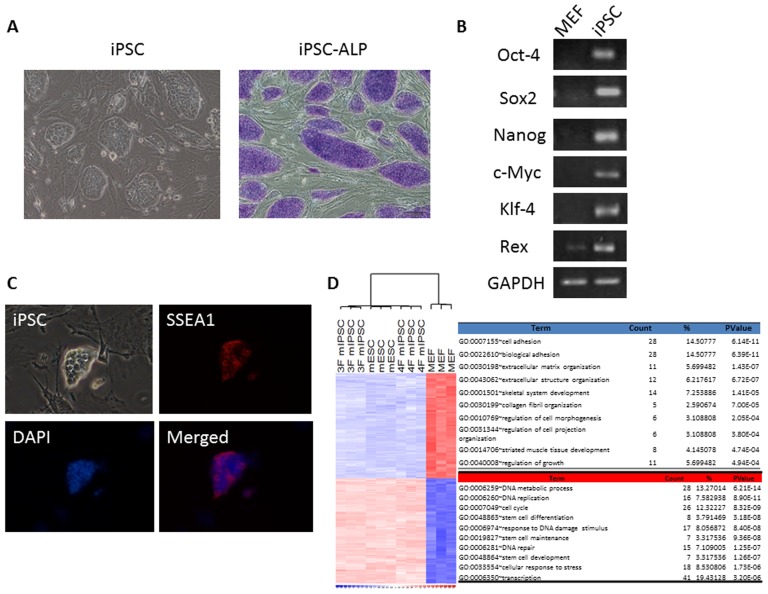
Characterization of three-gene mouse iPSCs. (A) Left: Three-gene mouse iPSCs were capable of forming colonies similar in appearance to that of ESCs. Right: Three-gene mouse iPSC colonies were positive for alkaline phosphate stain (Blue). (B) As compared to MEFs, the RT-PCR analysis showed that iPSCs expressed the stem cell gene markers (GAPDH: internal control). (C) These colonies of iPSCs were positive for SSEA-1 by immunofluorescent staining. (D) Gene expression microarray analysis showed that similar expression profiles among three-gene iPSCs, ESCs and four-gene iPSCs using a hierarchical heat map. DAPI = 4′,6-diamidino-2-phenylindole; ESCs = embryonic stem cells; iPSCs = induced pluripotent stem cells; iPSC-ALP = iPSC stained with alkaline phosphate; MEFs = mouse embryonic fibroblasts; mESC = mouse embryonic stem cell; 3F miPSC = three transcription factors without c-Myc mouse iPSC; 4F miPSC = four transcription factors mouse iPSC; SSEA-1 = stage-specific embryonic antigen 1.

In our present and previous studies [25], [27], we established pluripotent mouse-iPSCs without c-Myc and investigated molecular characteristics and the homing potential of transplanted iPSCs in the injured lung in mice with ALI (Figure 1 and Figure S1). We also explored the treatment effects of iPSCs and iPSC-CM and found that both iPSCs and iPSC-CM had similar effects in the improvement of mice with ALI (Figure S2). See supporting information file Text S1 for details.

### iPSCs or iPSC-CM attenuated high-tidal-volume-induced VILI

**Figure 2:**
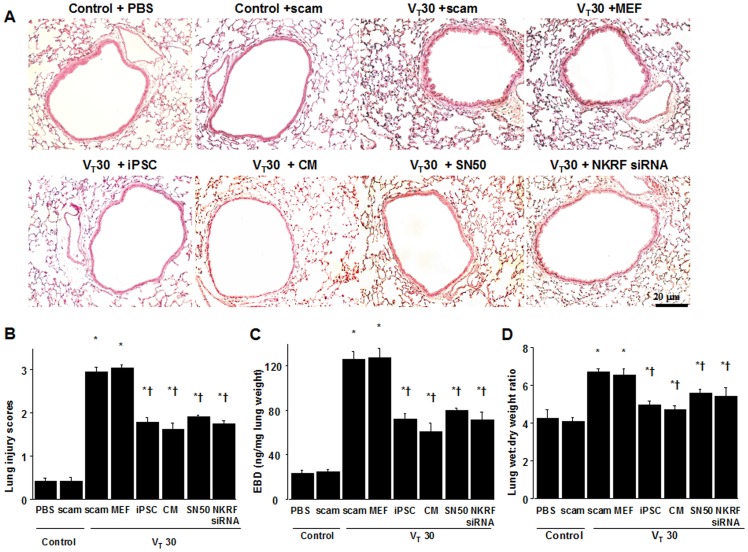
iPSCs or iPSC-CM, SN50, or NKRF siRNA reduced lung stretch-induced lung edema and injury. (A) Histological examination and (B) the quantification of high-tidal volume-induced airway structural damage and the restorative effect of iPSCs, iPSC-CM, SN50, or NKRF siRNA. The effects of administering iPSCs, iPSC-CM, SN-50, or NKRF siRNA on (C) lung EBD, and (D) the wet-to-dry ratio in wild-type mice receiving mechanical ventilation at a high-tidal-volume (V_T_30) are shown. SN50 (2 mg/kg) was given intraperitoneally 30 min before mechanical ventilation. NKRF siRNA, 6 mg/Kg, was given intratracheally 48 h before mechanical ventilation. Data shown here are the mean ± SD of five independent experiments. In panels B, C, and D, *P<0.05 vs. Non-ventilated control treated with PBS, †*P*<0.05 vs. V_T_30-ventilated mice treated with scam or MEF. Scale bars represent 20 µm. CM = conditioned medium of iPSCs; EBD = Evans blue dye; iPSCs = induced pluripotent stem cells; MEF = mouse embryonic fibroblasts; NKRF = NF-κB repressing factor; PBS = phosphate-buffered saline; Scam = nontargeting scrambled siRNA; siRNA = short interfering RNA.

We further employed high-tidal-volume (V_T_30 ml; denoted as V_T_30) ventilation with ambient air for 4 h to induce VILI in male C57BL/6 mice and examined the treatment effects of intravenously delivered iPSCs or iPSC-CM. Physiological conditions at the beginning and end of ventilation was shown in Table 1. Histological examinations revealed that the animal lungs injured by mechanical ventilation at V_T_30 displayed a pattern of alveolar congestion, hemorrhaging, thickening of the alveolar wall, and neutrophil infiltration, which were largely rescued by the administration of iPSCs or iPSC-CM (Figure 2A). The lung injury score quantification confirmed the V_T_30-induced severe damage and the therapeutic potential of iPSCs or iPSC-CM (Figure 2B). A V_T_30 also increased lung Evans blue dye (EBD) content and the wet-to-dry ratio, indicating capillary leakage. The microscopic lung congestion and elevation of capillary permeability induced by a V_T_30 was not affected by mouse embryonic fibroblast (MEF) treatment, but was substantially suppressed by treatment with either iPSCs or iPSC-CM (Figures 2C, 1D). Therefore, these data suggest that iPSCs or iPSC-CM improve microvascular leakage, lung edema, and total lung injury in a mouse VILI model induced by a V_T_30. The lung injury score quantification (Control, PBS = 0.46±0.07, Control, SN50 = 0.38±0.12, Control, NKRF siRNA = 0.42±0.08, Control, heat-inactivated iPSC-CM = 0.5±0.1, P = 0.51), levels of EBD (Control, PBS = 24.6±1.4 ng/mg lung weight, Control, SN50 = 22.2±1.3 ng/mg lung weight, Control, NKRF siRNA = 23.0±1.8 ng/mg lung weight, Control, heat-inactivated iPSC-CM = 25.3±1.7 ng/mg lung weight, P = 0.14), wet-to-dry weight ratio (Control, PBS = 4.2±0.5, Control, SN50 = 4.1±0.3, Control, NKRF siRNA = 4.2±0.2, Control, heat-inactivated iPSC-CM = 4.5±0.4, P = 0.41) were observed in control, nonventilated mice.

**Table 1:** Physiologic conditions at the beginning and end of ventilation.

	Control non-ventilated saline	V_T_ 30 ml/kg scam	V_T_ 30 ml/kg iPSCs	V_T_ 30 ml/kg CM	V_T_ 30 ml/kg SN50	V_T_ 30 ml/kg NKRF siRNA
PH	7.40±0.05	7.35±0.07	7.36±0.08	7.35±0.06	7.37±0.09	7.37±0.07
PaO_2_ (mmHg)	94.5±5.2	86.8±5.2	92.0±5.3	92.5±4.9	92.3±5.0	91.9±5.7
PaO_2_/FiO_2_ ratio	452.3±8.7	413.4±7.9	438.3±8.5	440.5±8.1	439.4±6.2	437.6±7.8
PaCO_2_ (mmHg)	39.2±0.3	36.0±1.2	35.9±1.0	36.1±0.8	36.1±1.3	36.4±0.5
MAP (mmHg)						
Start	84±1.0	82.7±1.3	82.5±1.2	82.3±1.3	82.1±1.4	82.4±0.6
End	82±0.7	76.8±4.1	77.6±4.1	77.8±4.5	77.8±3.7	78.7±0.9
PIP, mm Hg						
Start		23.4±1.2	23.7±1.4	23.5±1.3	23.6±1.2	23.4±1.3
End		26.8±2.3	25.7±2.6	25.8±2.9	25.9±2.5	25.7±1.9

Arterial blood gases and mean arterial pressure were obtained from non-ventilated mice and mice ventilated at a tidal volume of 6 ml/kg or 30 ml/kg for 4 h (n = 10 per group). No statistical difference was found in pH, PaO_2_, PaCO_2_, mean arterial pressure, and peak inspiratory pressure at the beginning versus the end of 4 h of mechanical ventilation. The normovolemic statuses of mice were maintained by monitoring mean artery pressure. Because no differences were observed between control, non-ventilated mice with or without iPSCs, with or without nontargeting scrambled siRNA, with or without SN50, with or without NKRF siRNA, with or without heat-inactivated conditioned medium, mice ventilated at 30 ml/kg with or without mouse embryonic fibroblasts, mice ventilated at 30 ml/kg with iPSCs or conditioned medium, the data were combined. CM = conditioned medium of iPSCs; FiO_2_ = fraction of inspired oxygen; iPSCs = induced pluripotent stem cells; MAP = mean arterial pressure; NKRF siRNA = nuclear factor-κB repressing factor short interfering RNA; PIP = peak inspiratory pressure; SN50 = NF-κB inhibitor; Scam = nontargeting scrambled siRNA; V_T_ = tidal volume. The physiological data of control groups were similar during the experiment and were used as the beginning data of ventilation.

### Reduced VILI-associated inflammatory response by iPSCs or iPSC-CM

**Figure 3:**
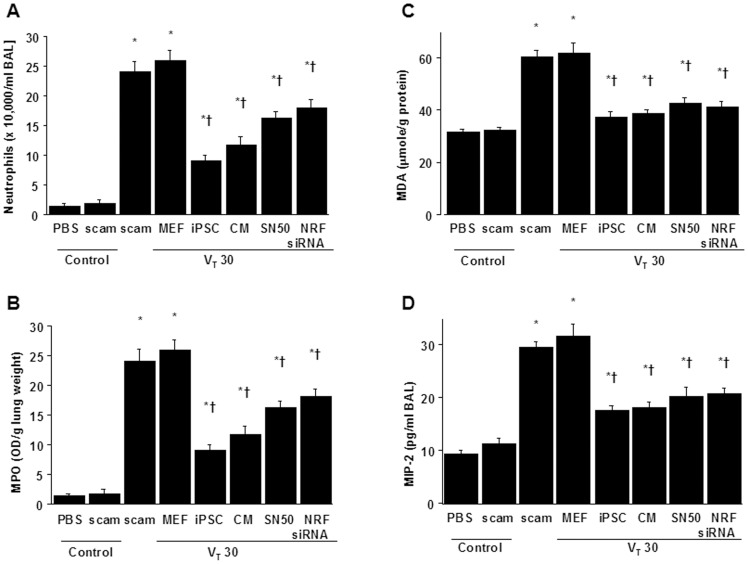
iPSCs or iPSC-CM, SN50, or NKRF siRNA attenuated lung stretch-induced neutrophil infiltration and oxidant stress. The effects of administering iPSCs, iPSC-CM, SN50, or NKRF siRNA on (A) neutrophil infiltration, (B) MPO activity, (C) MDA activity, and (D) MIP-2 secretion in BAL fluid in wild-type mice receiving mechanical ventilation at a high tidal-volume (V_T_30) are shown. Data shown here are the mean ± SD of five independent experiments. *P<0.05 vs. Non-ventilated control treated with PBS, †*P*<0.05 vs. V_T_30-ventilated mice treated with scam or MEF. BAL = bronchoalveolar lavage fluid; MDA = malondialdehyde; MIP-2 = macrophage inflammatory protein-2; MPO = myeloperoxidase.

We next identified neutrophils, the main inflammatory cells involved in the process of ALI [25]. The neutrophil counts and myeloperoxidase (MPO) assay revealed that neutrophils migrated into the injured lung sites in mice after mechanical ventilation at V_T_30 when compared with non-ventilated mice (Figures 3A, 3B). Meanwhile, malondialdehyde (MDA) level, which is an aldehydic secondary product of lipid peroxidation produced by neutrophils, and MIP-2 protein levels were elevated in response to V_T_30 treatment (Figures 3C, 3D), indicating an increase of oxidative stress and upregulation of chemoattractants for neutrophils in this model. Significantly, iPSCs or iPSC-CM ameliorated neutrophil migration, MDA, and MIP-2 protein elevation (Figures 3C, 3D). These data demonstrate that either iPSCs or iPSC-CM can attenuate neutrophil infiltration and inflammatory responses in high- tidal-volume-induced VILI.

### High-tidal-volume ventilation induced increased microvascular permeability, inflammation, and lung injury via the activation of NF-κB and NKRF, which were inhibited by SN50 and NKRF siRNA respectively

**Figure 4:**
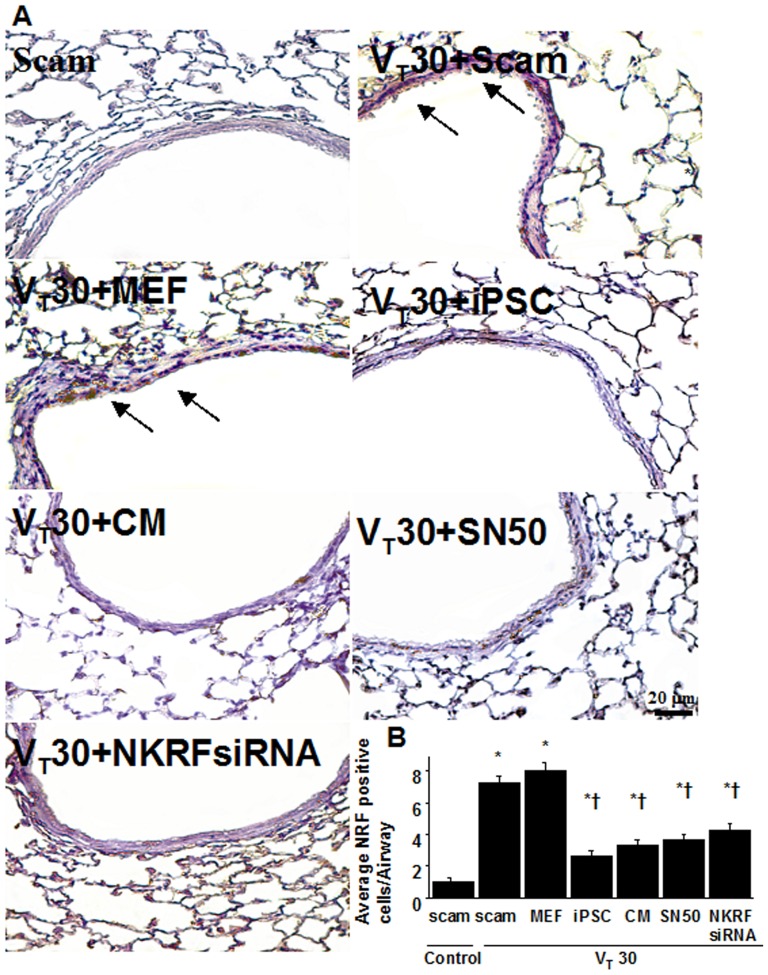
iPSCs or iPSC-CM, SN50, or NKRF siRNA abrogated lung stretch-induced NKRF expression in airway epithelium. Immunohistochemistry (A, ×400) and quantification (B) of NKRF expression were from the lungs of V_T_30-ventilated wild-type mice receiving iPSCs, iPSC-CM, SN50, or NKRF siRNA. Data shown here are representative results of five independent experiments. A dark brown diaminobenzidine signal identified by arrows indicates positive staining for NKRF in the lung epithelium or interstitial, whereas shades of bluish tan signify nonreactive cells. Data shown here are the mean ± SD of five independent experiments. *P<0.05 vs. Non-ventilated control treated with scam, †*P*<0.05 vs. V_T_30-ventilated mice treated with scam or MEF. Scale bars represent 20 µm.

**Figure 5:**
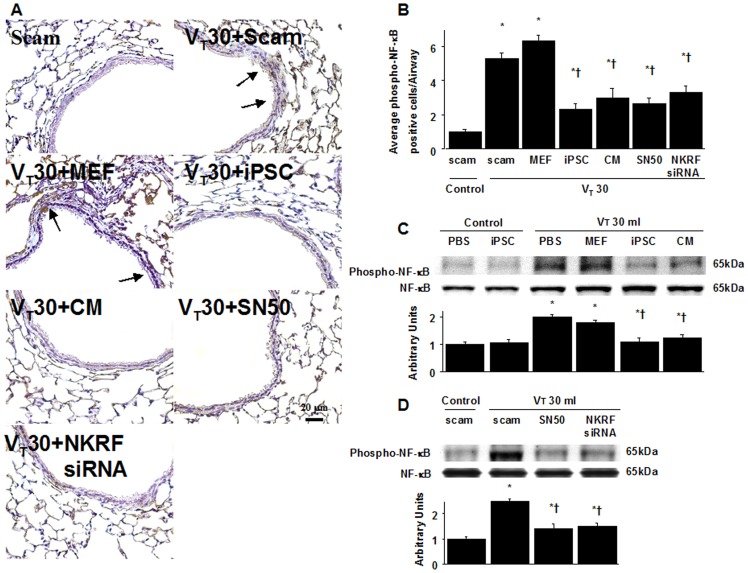
iPSCs or iPSC-CM, SN50, or NKRF siRNA suppressed lung stretch-induced NF-κB phosphorylation. NF-κB phosphorylation from the lungs of V_T_30-ventilated wild-type mice receiving iPSCs, iPSC-CM, SN50, or NKRF siRNA as detected by either (A, B) immunohistochemistry (×400) and quantification, (C, D) Western blot analyses. Data shown here are representative results of five independent experiments. Arbitrary units were expressed as the ratio of phospho-NF-κB to NF-κB. A dark brown diaminobenzidine signal identified by arrows indicates positive staining for phospho-NF-κB in the lung epithelium or interstitial, whereas shades of bluish tan signify nonreactive cells. Data shown here are the mean ± SD of five independent experiments. *P<0.05 vs. Non-ventilated control treated with scam, †*P*<0.05 vs. V_T_30-ventilated mice treated with scam, PBS, or MEF. Scale bars represent 20 µm.

**Figure 6:**
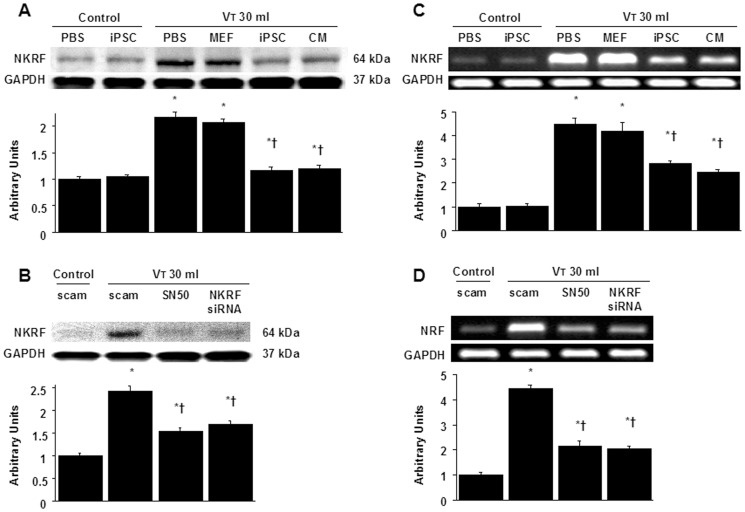
iPSCs or iPSC-CM, SN50, or NKRF siRNA abrogated lung stretch-induced NKRF and NKRF mRNA expression. NKRF expression from the lungs of V_T_30-ventilated wild-type mice receiving iPSCs, iPSC-CM, SN50, or NKRF siRNA was detected by Western blot analyses (A, B). The effects of administering of iPSCs, iPSC-CM, SN50, or NKRF siRNA on the mRNA expression of NKRF (C, D) are demonstrated. Data shown here are representative results of five independent experiments. Arbitrary units were expressed as the ratio of NKRF to GAPDH or NKRF mRNA to GAPDH. Data shown here are the mean ± SD of five independent experiments. *P<0.05 vs. Non-ventilated control treated with PBS or scam, †*P*<0.05 vs. V_T_30-ventilated mice treated with scam, PBS, or MEF.

NF-κB and NKRF have been shown to modulate the neutrophil activation involved in ALI [10], [18]. Immunohistochemistry indicated that the airway epithelium stained positive for NKRF and phospho-NF-κB after mechanical ventilation at V_T_30 (Figures 4A, 4B, 5A, 5B). To further investigate the interrelationship between NF-κB and NKRF in this VILI model, we next used NKRF siRNA or pharmacological NF-κB inhibition to identify the involvement of the NF-κB/NKRF pathway in high-tidal-volume-induced VILI. Consistent with the immunohistochemical findings, Western blot analyses revealed that NKRF expression and phospho-NF-κB phosphorylation were increased in mice receiving mechanical ventilation at V_T_30 and that NKRF knockdown and inhibiting NF-κB with SN50 abolished the V_T_30-induced NKRF and phospho-NF-κB activation (Figures 5C, 5D, 6A, 6B). NKRF knockdown and NF-κB inhibition also prevented NKRF mRNA upregulation in response to V_T_30 (Figures 6C, 6D). The administration of SN50 or NKRF siRNA also abrogated the lung injury scores, microvascular permeability, neutrophil influx, and the production of MDA and MIP-2 (Figures 2 and 3). Consistent with previous reports in ALI [10], [18], our data indicate that NF-κB/NKRF signaling is also required for the induction of VILI.

### Inhibition of high stretch ventilation-induced NF-κB/NKRF pathways by iPSCs or iPSC-CM, which was mimicked by pharmacological inhibition of NF-κB activity with SN50 or NKRF expression with NKRF siRNA

We next investigated whether the beneficial effects provided by iPSCs or iPSC-CM were mediated through NF-κB/NKRF pathways. We identified that the administration of iPSCs or iPSC-CM abolished the V_T_30-induced activation of NKRF mRNA and their protein production, which were measured by gene expression and western blot analyses (Figures 6A, 6B, 6C, 6D). In addition, either iPSCs or iPSC-CM reduced the V_T_30-induced phosphorylation of NF-κB, as measured by western blot analyses (Figure 5C, 5D). Furthermore, the positive immunohistochemical staining for NKRF or phospho-NF-κB in the lung epithelium or interstitial in mice at V_T_30 was significantly attenuated by the treatment of iPSCs or iPSC-CM (Figures 4A, 4B and 5A, 5B). These results indicate both iPSCs and iPSC-CM possess the abilities of suppressing the V_T_30-induced oxidative burst and inflammatory responses through the inhibition of NF-κB/NKRF pathways, which is mimicked by pharmacological inhibition of NF-κB activity with SN-50 or NKRF expression with NKRF siRNA as aforementioned.

### Ultramicrostructural restoration by iPSCs, iPSC-CM, SN50 or NKRF siRNA

**Figure 7:**
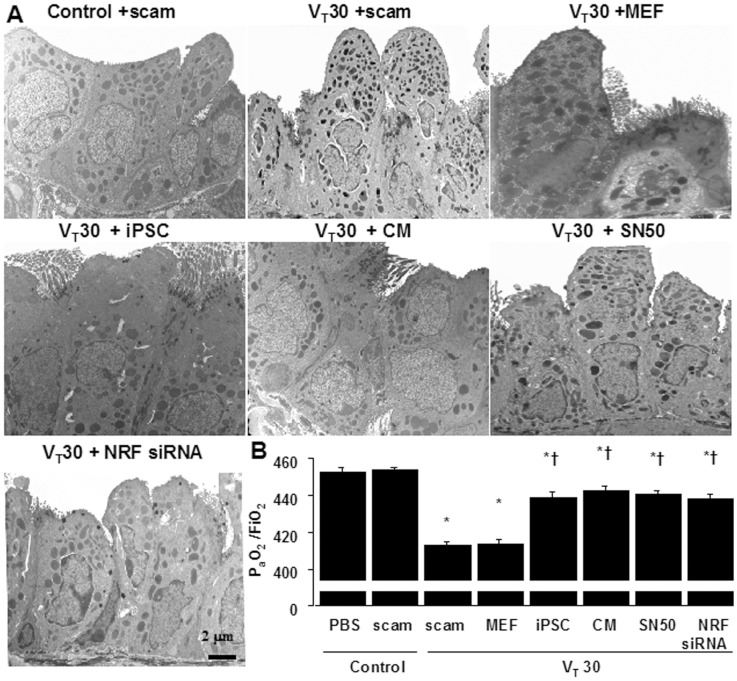
Restoration of ultramicrostructure and oxygenation by iPSCs or iPSC-CM, SN50, or NKRF siRNA. (A) A transmission electron microscopic image revealing the restoration of airway ultramicrostructure by iPSCs, iPSC-CM, SN50, or NKRF siRNA. (B) Restoration of oxygenation by iPSCs, iPSC-CM, SN50, or NKRF siRNA in wild-type mice receiving high-tidal-volume ventilation was shown. Data shown here are the mean ± SD of five independent experiments. *P<0.05 vs. Non-ventilated control treated with PBS, †*P*<0.05 vs. V_T_30-ventilated mice treated with scam or MEF. Scale bars represent 2 µm.

Transmission electron microscopy (TEM) was used to determine the effects of mechanical ventilation and iPSC therapy on the ultrastructures of bronchial epithelia (Figure 7A). The administration of V_T_30 led to disruption of the airway ultramicrostructures: reduced and indistinct microvilli, increases of secretary vesicles, and shrinkage of nuclei. Administration of iPSCs or iPSC-CM, similar to SN50 or NKRF siRNA, consistently restored the airway ultrastructural integrity in mice subjected to high V_T_ ventilation, which were actually demonstrated by TEM. Furthermore, the PaO2/FiO2 ratio, an index of functional gas exchange, was significantly deteriorated with a V_T_30 when compared with non-ventilated mice (Figure 7B). Remarkably, the decreases in oxygenation with a V_T_30 were significantly improved by the administration iPSCs, iPSC-CM, as well as SN50 or NKRF siRNA.

## Discussion

High-tidal-volume ventilation in healthy mice has been used to mimic the overdistension of the less injured and thus more compliant areas of lung found in ARDS. Previous studies demonstrated that hyperexpansion of the lung was the mechanism of volutrauma in VILI [1]–[3]. The changes of microvascular permeability were associated with severe epithelial and endothelial injury caused by recruitment of inflammatory cells and the production of soluble factors related with the biotrauma of lung. Although lung-protective ventilation strategy is beneficial, the mortality of ARDS has remained substantially high [28]. Therefore, novel therapies including cell-based therapy are needed to further reduce morbidity and mortality from this syndrome. The mechanisms of treatment with iPSCs or their soluble factors are not completely understood and need to be investigated in preclinical studies and further clinical trials. In our previous study, we observed the beneficial effects of iPSCs or iPSC-CM on the LPS-induced ALI in mice [25]. In this mouse VILI model, we have demonstrated that high V_T_ ventilation increased lung edema, microvascular permeability, neutrophil infiltration, production of MIP-2 of BAL fluid, intracellular oxidative stress, and total lung injury. Importantly, either iPSCs or iPSC-CM could protect mice against high stretch ventilation-induced lung injury and restore the functional gas exchange by improving oxygenation. We further explored the roles of NF-κB and NKRF in mediating the beneficial effects provided by iPSCs or iPSC-CM in VILI and found iPS cell-based therapy reduced high V_T_ mechanical ventilation- induced oxidative stress and inflammatory response through inhibiting NF-κB/NKRF-MIP-2(IL-8) signaling.

Stem cell therapy with iPSCs has been established to show efficacy in the treatment of cerebral stroke, spinal cord injury, and myocardial infarction [24], [29]–[30]; however, the effects of iPSCs on the ALI remain unknown. Injurious stimulus in the lung induces release of stromal cell-derived factor (SDF)-1a, secondary lymphoid chemokine, granulocyte colony-stimulating factor (G-CSF), cysteine-amino-cysteine receptor (CXCR) 4, and CXCR7, which stimulate homing of iPSCs to the damaged tissue [31]. The beneficial effects of iPSCs derive not only from the plasticity of engraftment in the lungs but also from their ability to secrete paracrine factors, which regulate endothelial and epithelial permeability, inflammation and repair. In our previous study of LPS-induced ALI in mice, we showed that iPSC therapy reduced the neutrophil influx in a cell-contact independent manner [25]. Because the time for which iPSCs differentiate to multipotent lung and airway progenitors would need for more than one week [32], [33] and there are only 4% of engraftment rate of IPSCs in the injured lung in mice with ALI, it's the effects of soluble factors in the iPSC-CM that modulates the process of attenuation in a cell-contact independent style [25]. Accumulating studies of LPS-induced ALI in mice treated with bone marrow-derived mononuclear cells or MSCs demonstrated that improvement of lung damage may be attributed to paracrine effects, despite lower percentage of stem cells engrafted into the lung [34]–[37]. Here we demonstrated that the iPSC-CM treatment was equally effective in reversing the high-tidal-volume-induced lung inflammation, microvascular leakage, and pathologic lung injury as iPSCs. Given that iPSC-CM containing soluble factors can be used to reduce the VILI as iPSCs by its paracrine effect, and iPSC-CM is considered to replace stem cells for avoiding the high risk of teratoma formation following stem cell transplantation. Otherwise, in this study we made refinement of our iPSCs procedure to remove oncogene by generating ectopic transfection of reprogramming factors Oct4/Sox2/Klf4 without c-Myc as previously described [27], and thus the potential for these cells used as a clinical therapeutic source becomes promising.

In the present study, we exhibited that high V_T_ ventilation recruited the influx of neutrophils as measured by infiltrating neutrophils of BAL fluid and total neutrophil sequestration by MPO levels of lung tissues, and increased the concentration of MDA, an aldehydic secondary product of lipid peroxidation used as a marker of oxidative damage. Neutrophils, mostly chemoattracted by MIP-2, are the major inflammatory cells involved in the process of VILI, and play a major role in the generation of enormous reactive oxygen species (ROS) [38]–[40]. NF-κB can be activated as an oxidative stress-responsive transcription factor required for maximal expression of many cytokines involved in the pathogenesis of ALI. In addition, binding elements for NF-κB are present in the promoter regions of cytokine genes, such as MIP-2 (IL-8) [41]. Previous studies indicated that administration of steroid blocking NF-κB or specific NF-κB inhibitor SN50 was proved to improve microvascular permeability, neutrophil influx into the alveolar lumen, and proinflammatory cytokines during VILI [10], [13]. Here, we have shown that both iPSCs and iPSC-CM could reduce the high V_T_ ventilation-induced NF-κB activation, thus prevented neutrophil influx into the alveolus, declined the amount of MIP-2 and MDA, attenuated alveolar epithelial capillary leak, and ameliorated the VILI. In consistent with our results, Yang el al. described that bone marrow stromal cell transplantation could protect against paraquat-induced acute lung injury by decreasing NF-κB activation [42] and Yagi et al. demonstrated that human MSCs have the capacity to inhibit the activation of NF-κB under LPS-stimulated inflammatory conditions [43]. Taken together, we have demonstrated iPSCs and iPSC-CM have the anti-oxidant and anti-inflammatory abilities to reduce the VILI through attenuating oxidative stress and inhibiting the activation of NF-κB and its downstream inflammatory mediators.

Next, we further investigated whether NKRF operated as a coactivator or repressor regulating NF-κB in the setting of VILI. IL-8 acts as a major mediator of acute neutrophil-mediated inflammation in response to mechanical cellular stress. The promoter of IL-8 gene contains NF-κB element that is required for activation in all cell types studied but enhanced NF-κB-induced transcriptional activity might additionally require phosphorylation of the subunits as well as binding of coactivators [44]. NKRF, a ubiquitously expressed nuclear transcription factor, binds to NREs of IL-8 promoter to specifically inhibit the transcriptional activity of NF-κB proteins. As noted, in IL-8 gene expression, NKRF exclusively was found to play a dual function. It represses the basal transcription of IL-8 gene in unstimulated cells. Together with NF-κB p65, NKRF acts as a coactivator to stimulate the IL-8 gene expression in IL-1-treated human epithelial cells [15]. Since IL-8 has been comprehensively studied as an NKRF-controlled gene, we therefore studied the interaction of NKRF with NF-κB in the regulation of MIP-2 production in VILI. We exhibited that both NKRF and NF-κB were activated in high stretch ventilation individually. Administering either using SN50 or short interfering RNA of NKRF could attenuate the high V_T_ ventilation-induced ALI. These results indicated the cooperation of NKRF with NF-κB, rather than repression, contributed to the downstream inflammatory cascades induced by high stretch ventilation, and this finding is in agreement with the dual role of NKRF in regulating IL-8 transcription. It is likely that NKRF may have dual roles in regulation of NF-κB signaling pathway, depending on the various experimental models e.g., chronic inflammatory airway diseases *vs.* acute lung injury, and different cell types e.g., airway smooth muscle cells *vs.* airway epithelial cells, location and timing of stimuli [45], [46]. In this study, we further demonstrated that iPSCs or iPSC-CM could decrease the high-tidal-volume induced coactivation of NF-κB and NKRF similar as SN50 or RNA interference of NKRF, thus reduced the subsequent oxidative stress and inflammatory responses drastically.

Because iPSCs can be derived from a patient's own somatic cells, they possess advantages to avoid immune rejection after transplantation and the ethical concerns raised by ESCs for the future clinical use purpose [20]–[23]. In contrast to ESCs and iPS cells, MSCs have a limited life span and become senescent and have risk of contamination when cultured *in vitro* [34]. Thus, iPSCs seem to be a promising stem cell source for autologous cell transplantation for specific diseases. However, the optimal route of cell delivery remains to be determined. Previous studies of stem cell therapy in mice showed the tumorigenicity by direct application of human ESCs [47] and temper the application of ESCs. Our previous studies revealed that it is a safe way to deliver iPSCs intravenously through tail vein and no teratoma formation was observed after 90 days of follow-up [25]. Because high stretch ventilation-induced biotrauma and subsequent multiple organ involvement can be accompanied by VILI, intravenous route used in this study might be the preferred approach to exert the systemic beneficial effect as noted [48]. Nevertheless, the risk of teratoma formation is still a concern in stem cell therapy, and the long term follow-up of iPSC therapy and modification of refined iPSCs procedure are required in the future study.

Our study had limitations. Stem cells can escape clearance by the host immune system through mechanisms including low expression of the major histocompatibility complex (MHC) I and II proteins as well as lack of the T-cell costimulatory molecules, CD40, CD80 and CD86 [49]. However, recent studies showed that MSC can express higher levels of the MHC class proteins and may induce a host response and lead to graft rejection [50], [51]. The immunological privilege about iPSCs should be carefully clarified. Second, MSCs possess potent immunosuppressive effects mediated by cell contact-dependent and cell contact-independent mechanisms through the release of soluble factors, including IL-10, prostaglandin E2, keratinocyte growth factor, granulocyte colony-stimulating factor, and IL-1 receptor antagonist [35], [49]–[51]. The cytokine arrays and quantitative analyses for soluble mediators of iPSC-CM will help us to identify these useful mediators in the stem cell therapy. Third, Lee et al. described the therapeutic potential of MSCs in two human models of ALI including an *ex vivo* human lung preparation and primary cultures of human alveolar epithelial type II cells injured by inflammatory insults [49]. But the application of personalized iPSC therapy in critically ill patients with ALI necessitates the technological innovation such as fast harvesting and rapid expansion of transplanted iPSCs. Clinical trials will be imperative to determine the realistic effects of iPSC-based therapy in the acute and reparative phases of patients with ARDS using ventilator support in the near future. The pathways involved in endotoxin- and ventilator-induced lung injury are different. In the induction of lung injury by LPS, the increases of lung injury by LPS induces a generalized inflammation primarily in the endothelial cells of the lung; however, ventilator-induced mechanical stretch lead to the destabilization of alveolar-epithelial and capillary-endothelial barriers thereby resulting in increased vascular permeability and pulmonary edema [52].

## Conclusions

The National Heart, Lung and Blood Institute working group on ARDS identified to explore the molecular basis of mechanical stress-induced lung injury as a fertile area of future research, because VILI may lead to systemic cytokines translocation and multiple organ failure in clinical setting [53]. By using an *in vivo* mouse VILI model, we demonstrated high V_T_ ventilation-induced lung injury was associated with neutrophil influx, oxidative stress, alveolar epithelial- capillary damage, and production of MIP-2. These severe inflammation, edema, pathologic destruction, and impaired gas exchange of injured lungs were attenuated by either intravenous iPSCs or iPSC-CM and were, at least in part, mediated by inhibiting the NF-κB/NKRF pathways. Notably, iPSC-CM revealed comparable effects equal to those of iPSCs and the conditioned medium containing soluble factors deserves further investigations. Understanding the beneficial effects of iPSC therapy related with the suppression of NF-κB/NKRF signaling pathway and inflammatory responses may allow clarification of the biomolecular mechanisms regulating VILI and provide insight into novel therapeutic option for ALI/ARDS.

## Materials and Methods

### Ethics of experimental animals

Male C57BL/6 mice, aged between 6 and 8 weeks, weighing between 20 and 25 g, were obtained from the National Laboratory Animal Center (Taipei, Taiwan). The study was performed in strict accordance with the recommendations in the Guide for the Care and Use of Laboratory Animals of the National Institutes of Health. The protocol was approved by the Institutional Animal Care and Use Committee of Chang Gung Memorial Hospital (Permit Number: 2011093005). All surgery was performed under ketamine and xylazine anesthesia, and all efforts were made to minimize suffering. The experimental group of animals and procedures used in this study is summarized in Table 2.

**Table 2:** Experimental design and numbers of animals per group.

	EBD, lung edema, ROS (4 h)	MIP-2, neutrophils, MPO (4 h)	NF-κB/NKRF protein, NKRF mRNA (1 h)	IHC assay, lung injury score (4 h)	TEM (4 h)
Control+PBS	5	5	5	5	3
Control+scam	5	5	5	5	3
Control+iPSCs	5	5	5	5	3
Control+SN50	4	0	0	4	0
Control+NKRF siRNA	4	0	0	4	0
Control+heat-inactivated CM	4	0	0	4	0
V_T_ 6 ml+PBS	5	5	5	5	3
V_T_ 30 ml+PBS	5	5	5	5	3
V_T_ 30 ml+MEF	5	5	5	5	3
V_T_ 30 ml+iPSCs	5	5	5	5	3
V_T_ 30 ml+CM	5	5	5	5	3
V_T_ 30 ml+SN50	5	5	5	5	3
V_T_ 30 ml+NKRF siRNA	5	5	5	5	3

Control = spontaneously breathing, non-ventilated mice; CM = conditioned medium of iPSCs; EBD = Evans blue dye; TEM = transmission electron microscopy; IHC = immunohistochemical stain; iPSCs = induced pluripotent stem cells; MEF = mouse embryonic fibroblast; MIP-2 = macrophage inflammatory protein-2; MPO = myeloperoxidase; NF-κB = nuclear factor-κB; NKRF = NF-κB repressing factor; ROS = reactive oxygen species; PBS = phosphate-buffered saline; Scam = nontargeting scrambled siRNA; siRNA = short interfering RNA; V_T_ = tidal volume.

### Ventilator protocol

We used our established mouse model of VILI, as previously described [5]. In brief, a tracheostomy was performed under general anesthesia with intraperitoneal ketamine (90 mg/kg) and xylazine (10 mg/kg), followed by ketamine (0.1 mg/g/h) and xylazine (0.01 mg/g/h) at a rate of 0.09 ml/10 g/h by a continuous intraperitoneal infusion in male C57BL/6 mice. The mice were placed in a supine position on a heating blanket and then attached to a Harvard apparatus ventilator, model 55-7058 (Harvard Apparatus, Holliston, MA), which were programmed to administer 30 ml/kg at a rate of 65 breaths per min, for 1 to 4 h while breathing ambient air with zero end-expiratory pressure. The tidal volume delivered by the ventilator was confirmed by fluid displacement from an inverted calibration cylinder. The continuous monitoring of end-tidal CO_2_ with a microcapnograph (Columbus Instruments, Columbus, OH) was performed, and respiratory frequencies of 135 breaths per min for 6 ml/kg and 65 breaths per min for 30 ml/kg were selected with end-tidal CO_2_ at 30 to 40 mm Hg. The airway peak inspiratory pressure was measured with a pressure-transducer amplifier (Gould Instrument Systems, Valley View, OH) connected to the tubing at the proximal end of the tracheostomy. The mean arterial pressure was monitored each hour during mechanical ventilation using the same pressure-transducer amplifier connected to a 0.61-mm outer diameter (0.28-mm inner diameter) polyethylene catheter ending in the common carotid artery. One hour of mechanical ventilation was employed for RT-PCR and Western blot analyses, and 4 h was applied for MIP-2 production, cell counts, lung water, Evans blue dye, myeloperoxidase, free radicals, electron microscopy, and histopathologic staining analyses, based on previous studies [5], [10]. The control, nonventilated mice were anesthetized and sacrificed immediately. At the end of the study period, heparinized blood was extracted from the arterial line for analyses of arterial blood gas, and the mice were then sacrificed.

### Mouse embryonic fibroblasts (MEF), iPSCs and conditioned medium

Murine-iPSCs were generated from non-reprogrammed MEFs derived from C57BL/6 mice. The iPSCs were reprogrammed by the transduction of retroviral vectors encoding four transcription factors, Oct-4, Sox2, and Klf4, as described previously [25], [27]. The MEFs (5×10^7^ cells/kg suspended in phosphate-buffered saline (PBS)), iPSCs (5×10^7^ cells/kg, suspended in PBS), conditioned medium (200 µ L) from iPSCs, or PBS (200 µ L) were injected via tail vein 1 h before mechanical stretch based on our present and previous dose-response studies that showed 5×10^7^ cells/kg iPSCs and 200 µ L iPSC-CM improved lung injury and ameliorated neutrophil influx ([25] and Figure S3). For heat inactivation, conditioned media were treated at 95°C for 15 min [54]. Details are provided in supporting information file Text S1.

### Short interfering RNA administration

A single dosage of 6 µg/g of targeting NKRF Stealth siRNA or non-targeting Stealth siRNA (scrambled siRNA, Invitrogen, Carlsbad, CA) in lipofectamine RNAiMAX was given intratracheally 48 h before mechanical ventilation. The dosage was chosen on the basis of our dose-response studies that showed 6 µg/g inhibited NKRF activity.

### Pharmacological inhibitor

NF-κB inhibitor (SN-50, Calbiochem, San Diego, CA) 2 µg/g in PBS was given intraperitoneally 30 min before ventilation based on our dose-response studies that showed 2 µg/g inhibited NF-κB activity [10].

### Measurement of malondialdehyde

The lungs were homogenized in phosphate buffered saline containing butylated hydroxytoluene. The MDA in the protein extracts was measured using the Oxiselect TBARS assay kit (Cell Biolabs, San Diego, CA) containing thiobarbituric acid reactive substances. Each sample was run in duplicate and expressed as µmole/g protein according to the manufacturer's instructions.

### Immunoblot analysis

The lungs were homogenized in 3 ml of lysis buffer (20 mM HEPES pH 7.4, 1% Triton X-100, 10% glycerol, 2 mM ethylene glycol-bis (β-aminoethyl ether)-N, N, N′, N′-tetraacetic acid, 50 µM β-glycerophosphate, 1 mM sodium orthovanadate, 1 mM dithiotreitol, 400 µM aprotinin, and 400 µM phenylmethylsulfonyl fluoride), transferred to eppendorff tubes and placed on ice for 15 min. Tubes were centrifuged at 14,000 rpm for 10 min at 4°C and supernatant was flash frozen. Crude cell lysates were matched for protein concentration, resolved on a 10% bis-acrylamide gel, and electrotransferred to Immobilon-P membranes (Millipore Corp., Bedford, MA). For assay of NF-κB phosphorylation and total NF-κB and NKRF protein expression, Western blot analyses were performed with antibodies of phospho-NF-κB, NF-κB, and NKRF (Santa Cruz Biotechnology, Santa Cruz, CA). Blots were developed by enhanced chemiluminescence (NEN Life Science Products, Boston, MA).

### Reverse transcription-polymerase chain reaction

For isolating total RNA, the lung tissues were homogenized in TRIzol reagents (Invitrogen Corporation, Carlsbad, CA) according to the manufacturer's instructions. Total RNA (1 µg) was reverse transcribed by using a GeneAmp PCR system 9600 (PerkinElmer, Life Sciences, Inc., Boston, MA), as previously described [3]. The following primers were used for PCR: NKRF, forward primer 5′-GTTCTGCCAAACACTGGACC-3′ and reverse primer 5′-CTGAGATAGGCTCCCGTATGCCC-3′ and GAPDH as internal control, forward primer 5′-AATGCATCCTGCA CCACCAA-3′ and reverse primer 5′-GTAGCCATATTCATTGTCATA-3′ (Integrated DNA Technologies, Inc., Coralville, IA) [14].

### Immunohistochemistry

The lungs were paraffin embedded, sliced at 4 µm, deparaffinized, antigen unmasked in 10 mM sodium citrate (pH 6.0), incubated with goat NKRF or rabbit phospho-NF-κB primary antibody (1∶100; Santa Cruz Biotechnology, Santa Cruz, CA), and biotinylated goat anti-rabbit secondary antibody (1∶100) according to the manufacturer's instruction for an immunohistochemical kit (Santa Cruz Biotechnology, Santa Cruz, CA).

### Microarray analysis and bioinformatics

Total RNA was extracted from cells using Trizol reagent (Life Technologies, Bethesda, MD, USA) and the Qiagen RNAeasy (Qiagen, Valencia, CA, USA) column for purification. Total RNA was reverse-transcribed with Superscript II RNase H-reverse transcriptase (Gibco BRL) to generate Cy3- and Cy5-labeled (Amersham Biosciences Co., Piscataway, NJ, USA) cDNA probes for the control and treated samples, respectively. The labeled probes were hybridized to a cDNA microarray containing 10,000 gene clone immobilized cDNA fragments. Fluorescence intensities of Cy3 and Cy5 targets were measured and scanned separately using a GenePix 4000B Array Scanner (Axon Instruments, Burlingame, CA, USA). Data analysis was performed using GenePix Pro 3.0.5.56 (Axon Instruments, USA) and GeneSpring GX 7.3.1 software (Agilent, Palo Alto, CA, USA). The average-linkage distance was used to assess the similarity between two groups of gene expression profiles as described below. The difference in distance between two groups of sample expression profiles to a third was assessed by comparing the corresponding average-linkage distances (the mean of all pair-wise linkages between members of the two groups concerned). The error of such a comparison was estimated by combining the standard errors (the standard deviation of pair-wise linkages divided by the square root of the number of linkages) of the average-linkage distances involved. Classical multidimensional scaling (MDS) was performed using the standard function of the R program to provide a visual impression of how the various sample groups are related.

### Histopathologic grading of VILI

The lung tissues from control, nonventilated mice and mice exposed to high tidal volume ventilation for 4 h while breathing room air were removed *en bloc* and filled with 10% neutral buffered formalin (pH 6.8 to 7.2) at 30-cm H_2_O pressure via polyethylene tubing inserted into the trachea. The lungs were paraffin embedded, sliced at 4 µm, stained with hematoxylin and eosin, and reviewed from 10 nonoverlapping fields by a single investigator blinded to therapeutic category of the mouse. Lung injury was scored using an average score of the following items: alveolar congestion, hemorrhage, infiltration of neutrophils into airspace or the vessel wall, and thickness of the alveolar wall [3]. A score of 0 represented normal lungs; 1, mild, <25% lung involvement; 2, moderate, 25% to 50% lung involvement; 3, severe, 50% to 75% lung involvement; and 4, very severe, >75% lung involvement.

### Transmission electron microscopy

The lungs were fixed in 3% glutaraldehyde in 0.1 M cacodylate buffer (pH 7.4) for 1 h at 4°C. The lungs were then postfixed in 1% osmium tetroxide (pH 7.4), dehydrated in a graded series of ethanol, and embedded in EPON-812. Thin sections (70 nm) were cut, stained with uranyl acetate and lead citrate, and examined on a Hitachi H-7500 EM transmission electron microscope (Hitachi, Ltd., Tokyo, Japan).

### Statistical evaluation

The Western blots and NKRF mRNA were quantitated using a National Institutes of Health (NIH) image analyzer, ImageJ 1.27z (National Institute of Health, Bethesda, MD, USA) and presented as arbitrary units. Values were expressed as the mean ± SD for at least five experiments. The data of Evans blue dye assay, lung wet-to-dry weight ratio, MIP-2, MPO, immunohistochemical assay, and MDA were conducted by using Statview 5.0 (Abascus Concepts Inc. Cary, NC, USA; SAS Institute, Inc.). All results of Western blots and NKRF mRNA were normalized to control, nonventilated mice with room air. ANOVA was used to assess the statistical significance of the differences, followed by multiple comparisons with a Scheffe's test, and a *P* value <0.05 was considered statistically significant. Regression coefficients were calculated using the simple regression test in Statview.

Analysis of lung water, cell counts, EBD analysis, MPO assay, and measurements of MIP-2 were performed as previously described [3], [5].

## Supporting Information

Figure S1 **High tidal volume ventilation increased iPSCs trafficked in the lung.** Representative photomicrographs (×400) with Hoechst (blue) immunofluorescent staining of frozen lung sections were from (A) control, non-ventilated mice and (B) mice ventilated at V_T_ 30 ml/kg for 4 h with room air. (C) The scattered density of the incorporated iPSCs in the lung was quantified as an average number of Hoechst-labeled iPSCs in 10 nonoverlapping fields of lung sections. Positive blue staining in the lung epithelium and interstitium is identified by arrows. The positive staining of Hoechst in the lung sections of mice increased after mechanical ventilation at V_T_ 30 ml/kg for 4 h compared with that of control, nonventilated mice. Data shown here are the mean ± SD of four independent experiments. *P<0.05 vs. Non-ventilated control treated with PBS. Scale bars represent 20 µm. iPSCs = induced pluripotent stem cells; PBS = phosphate-buffered saline.
(TIF)

Figure S2 **LPS-induced ALI in mice treated by iPSCs/iPSC-CM and MEF/MEF-CM.** We used the intratracheal injection of LPS in C57BL/6 mice to induce acute lung injury. To investigate the treatment effect of iPSCs and iPSC-derived conditioned medium, we further injected the iPSCs, MEF, iPSC-CM, and MEF-CM into the mice of LPS-induced ALI through tail vein. Our results showed that both iPSCs and iPSC-CM significantly improved the lung injury in LPS-induced ALI in mice as compared to those of MEF or MEF-CM-treated mice. Importantly, the results of microarray analysis showed that both iPSCs and iPSC-CM could modulate the similar gene cluster expression in the lung lesions of LPS-induced ALI mice, suggesting that there existed the common trait of the biomolecular signatures in response to LPS-induced lung injury between the iPSCs and conditioned medium of iPSCs. ALI = acute lung injury; iPSC-CM = the conditioned medium of iPSCs; LPS = lipopolysaccharide; MEF = mouse embryonic fibroblasts; MEF-CM: the conditioned medium of MEF.
(TIF)

Figure S3 **iPSCs or iPSC-CM dose-dependently attenuated high-tidal-volume-induced lung injury and neutrophil infiltration.** The effects of administering iPSCs or iPSC-CM on (A) the quantification of airway structural damage and (B) neutrophil infiltration in bronchoalveolar lavage fluid in wild-type mice receiving mechanical ventilation at a high tidal volume (V_T_30) are shown. Data shown here are the mean ± SD of four independent experiments. *P<0.05 vs. V_T_30-ventilated mice treated with PBS.
(TIF)

Text S1 (DOC)
