# Supplementary figures and images for: Suppressing NF-κB and NKRF Pathways by Induced Pluripotent Stem Cell Therapy in Mice with Ventilator-Induced Lung Injury
Source: PLoS One. 2013 Jun 26;8(6):e66760. doi: 10.1371/journal.pone.0066760 (PMC3694116; doi:10.1371/journal.pone.0066760)

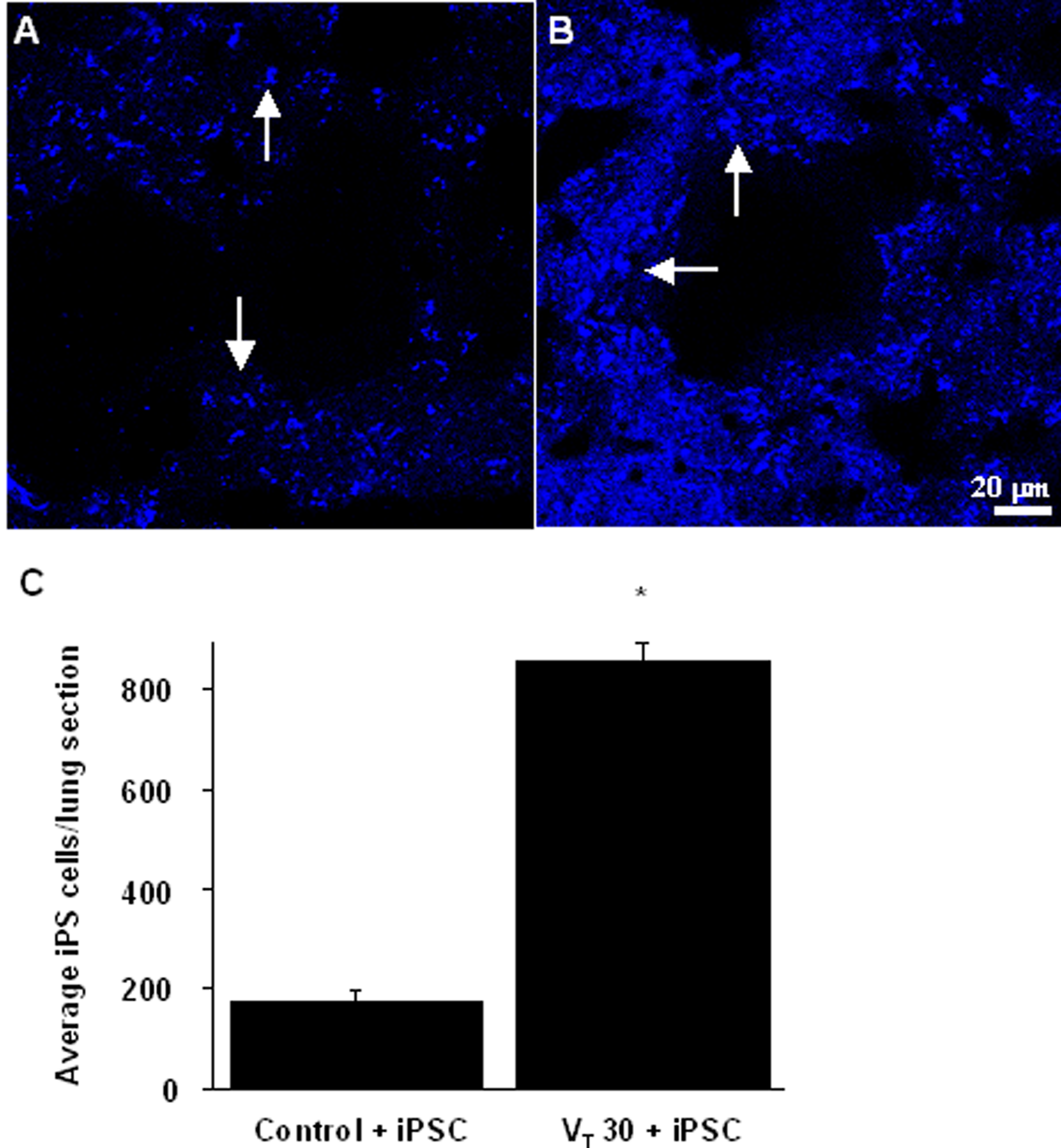

Supplement: Figure S1 — High tidal volume ventilation increased iPSCs trafficked in the lung. Representative photomicrographs (×400) with Hoechst (blue) immunofluorescent staining of frozen lung sections were from (A) control, non-ventilated mice and (B) mice ventilated at VT 30 ml/kg for 4 h with room air. (C) The scattered density of the incorporated iPSCs in the lung was quantified as an average number of Hoechst-labeled iPSCs in 10 nonoverlapping fields of lung sections. Positive blue staining in the lung epithelium and interstitium is identified by arrows. The positive staining of Hoechst in the lung sections of mice increased after mechanical ventilation at VT 30 ml/kg for 4 h compared with that of control, nonventilated mice. Data shown here are the mean ± SD of four independent experiments. *P<0.05 vs. Non-ventilated control treated with PBS. Scale bars represent 20 µm. iPSCs = induced pluripotent stem cells; PBS = phosphate-buffered saline. (TIF) [file pone.0066760.s001.tif]

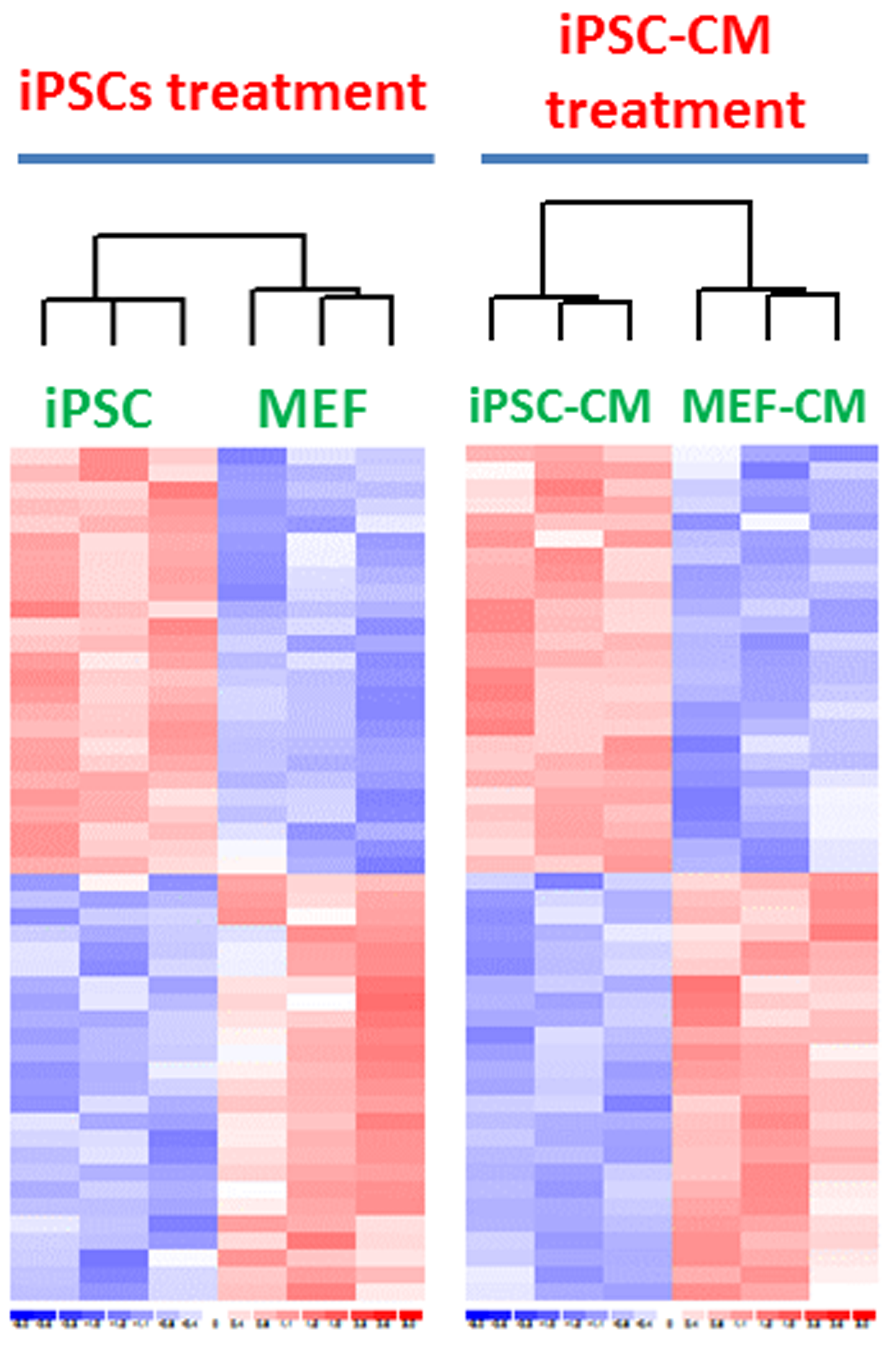

Supplement: Figure S2 — LPS-induced ALI in mice treated by iPSCs/iPSC-CM and MEF/MEF-CM. We used the intratracheal injection of LPS in C57BL/6 mice to induce acute lung injury. To investigate the treatment effect of iPSCs and iPSC-derived conditioned medium, we further injected the iPSCs, MEF, iPSC-CM, and MEF-CM into the mice of LPS-induced ALI through tail vein. Our results showed that both iPSCs and iPSC-CM significantly improved the lung injury in LPS-induced ALI in mice as compared to those of MEF or MEF-CM-treated mice. Importantly, the results of microarray analysis showed that both iPSCs and iPSC-CM could modulate the similar gene cluster expression in the lung lesions of LPS-induced ALI mice, suggesting that there existed the common trait of the biomolecular signatures in response to LPS-induced lung injury between the iPSCs and conditioned medium of iPSCs. ALI = acute lung injury; iPSC-CM = the conditioned medium of iPSCs; LPS = lipopolysaccharide; MEF = mouse embryonic fibroblasts; MEF-CM: the conditioned medium of MEF. (TIF) [file pone.0066760.s002.tif]

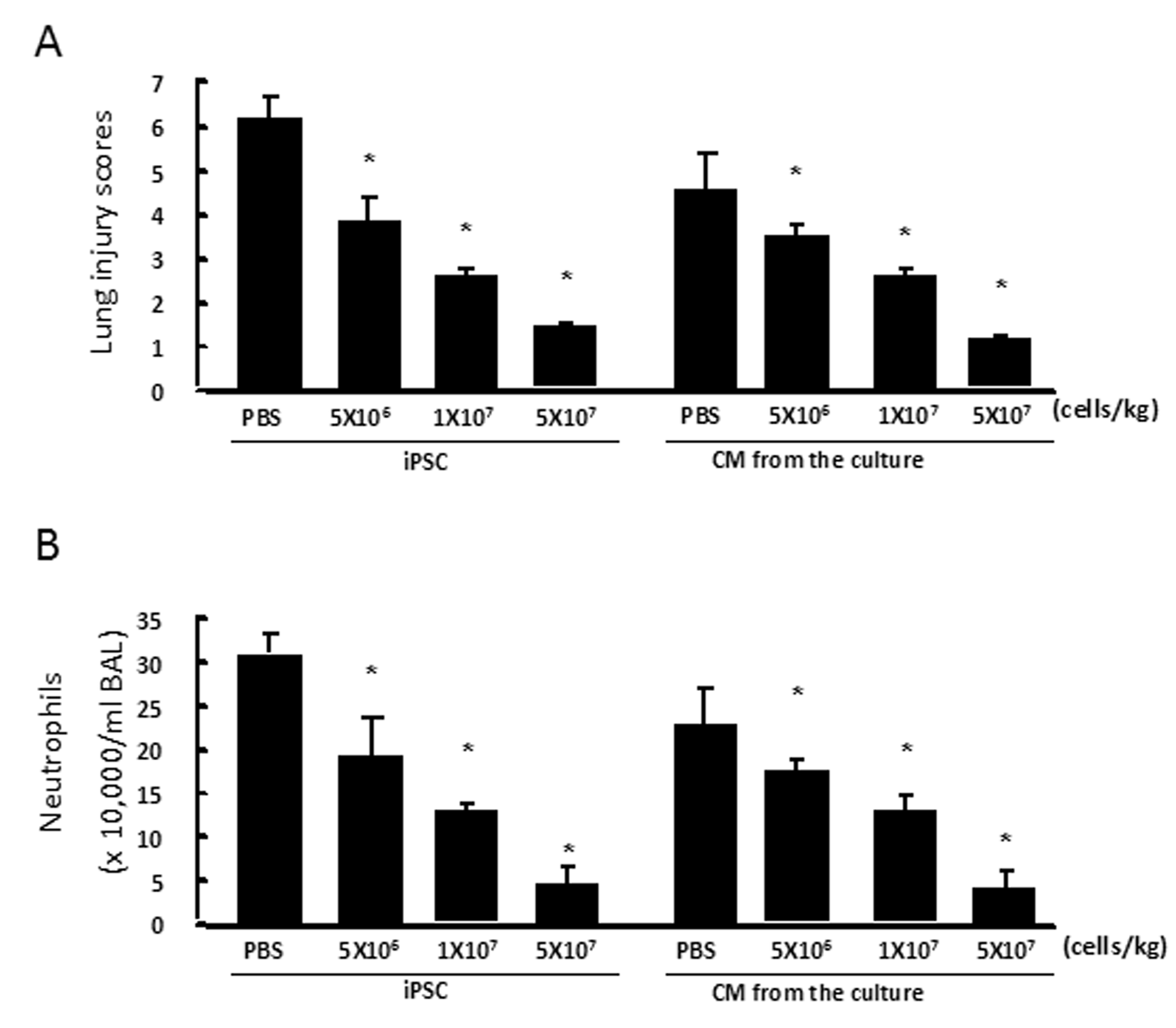

Supplement: Figure S3 — iPSCs or iPSC-CM dose-dependently attenuated high-tidal-volume-induced lung injury and neutrophil infiltration. The effects of administering iPSCs or iPSC-CM on (A) the quantification of airway structural damage and (B) neutrophil infiltration in bronchoalveolar lavage fluid in wild-type mice receiving mechanical ventilation at a high tidal volume (VT30) are shown. Data shown here are the mean ± SD of four independent experiments. *P<0.05 vs. VT30-ventilated mice treated with PBS. (TIF) [file pone.0066760.s003.tif]
