# Supplementary material for: Suppressing NF-κB and NKRF Pathways by Induced Pluripotent Stem Cell Therapy in Mice with Ventilator-Induced Lung Injury
Source: PLoS One. 2013 Jun 26;8(6):e66760. doi: 10.1371/journal.pone.0066760 (PMC3694116; doi:10.1371/journal.pone.0066760)
Supplement: Text S1 — (DOC) [file pone.0066760.s004.doc]

Suppressing NF-κB and NKRF Pathways by Induced Pluripotent Stem Cell Therapy in Mice with Ventilator-Induced Lung Injury

Yung-Yang Liu^1, 2^, Li-Fu Li ^3, 4, 5*^, Cheng-Ta Yang ^3, 4, 5^, Kai-Hsi Lu^6, 7^, Chung-Chi Huang ^3, 4, 5^, Kuo-Chin Kao ^3, 4, 5^, and Shih-Hwa Chiou^2, 8, 9, 10*^

The following data are supporting information file of PLoS One.

**Text S1**

**Materials and methods**

**Induced pluripotent stem cells (iPSCs) culture and in vitro differentiation**

Murine-iPSCs were generated from mouse embryonic fibroblasts (MEFs) derived from 13.5-day-old embryos of C57/B6 mice. The iPSCs were reprogrammed by the transduction of retroviral vectors encoding three transcription factors, Oct-4, Sox2, and Klf4, as described previously [1]. Briefly, undifferentiated iPSCs (20,000 cells/cm^2^) were routinely cultured and expanded on mitotically inactivated MEFs in six-well culture plates (BD Technology, Franklin Lakes, NJ) in the presence of 0.3% leukemia inhibitory factor (LIF) in an iPSCs medium consisting of Dulbecco's modified Eagle's medium (DMEM; Sigma, St. Louis, MO) supplemented with 15% fetal bovine serum (FBS; Invitrogen, Carlsbad, CA), 10mM non-essential amino acid (NEAA; Gibco), 0.1mM β-mercaptoethanol (Sigma), and 1% penicillin-streptomycin. The mouse iPSCs were transfected with pCX-EGFP to express green fluorescence constitutively and were maintained and differentiated in vitro as described previously [2]. Every three to four days, colonies were detached with 0.2% collagenase IV (Invitrogen), dissociated into single cells with 0.025% trypsin (Sigma-Aldrich, St. Louis, MO) and 0.1% chicken serum (Invitrogen) in phosphate-buffered saline (PBS), and plated again onto MEFs. For embryoid body (EB) formation, iPSCs were dissociated into a single cell suspension by 0.25% trypsin-EDTA and plated onto non-adherent culture dishes in DMEM with 15% FBS, 100 mM MEM non-essential amino acids, 0.55 mM 2-mercaptoethanol, and antibiotics at a density of 2×10^6^ cells/100-mm plate. After four days as a floating culture, EBs were transferred onto gelatin-coated plates and maintained in the same medium for 24 h. The selection of nestin-positive cells was initiated by replacing the medium with DMEM/F12 (Gibco) supplemented with Insulin-Transferrin-Selenium (ITS) (Gibco), 5 μg/ml fibronectin (Sigma), and antibiotics. After 6-10 days of selection, cells were trypsinized to remove remnant EBs and then plated onto glass coverslips or culture dishes precoated with polyornithine (15 μg/ml; Sigma) and fibronectin (1 μg/ml; Sigma or Gibco) at a concentration of 2×10^5^ cells/cm^2^. Neural precursor cells were expanded in N_2_ medium 3 containing 20 ng/ml basic FGF (bFGF), a murine N-terminal fragment of sonic hedgehog (SHHN), FGF8-b (all from R & D Systems), and 200 μM ascorbic acid (AA; Sigma) for 7 to 10 days.

**Preparation of the iPSC conditioned medium (iPSC-CM)**

Murine-iPSCs were placed at 20,000 cells per cm^2^ and incubated at the volume of 10 ml serum-free basal medium (DMEM-high glucose (Gibco), 10mM non-essential amino acid (NEAA; Gibco), 0.3% LIF, and 1% penicillin-streptomycin) in 10-cm dish (Corning Incorporated) for 48 h. Then the trypsinized iPSCs with whole culture medium were collected and centrifuged for 10 min at 1,500 rpm to obtain the supernatant as the conditioned medium. The conditioned medium was used for further *in vivo* experiments [3-5].

**Cell-labeling protocol**

Hoechst 33258 (Sigma Chemical, St. Louis, MO) is widely used to label DNA, and these fluorescent stains are commonly used to visualize nuclei. In serum-free culture medium, 5x10^7^ cells/kg mouse iPSCs were incubated with Hoechst 33258 (10 μM) for 60 min at 37°C, and then centrifuged at 1,500 rpm for 5 min at 37°C. Later, we removed the supernatant and gently re-suspended the cells in PBS. Hoechst-labeled-iPSCs were directly applied to animal experiments. The lungs from iPSCs pre-treated mice subjected to the high tidal volume ventilation were frozen in Tissue-Tek optimal cutting temperature (OCT) compound by immersion in liquid nitrogen. Cryostat sections (10 μm thick) were adhered to glass slides previously coated with poly-L-lysine, fixed in methanol for 5 min. The fluorescence-labeled slides were then examined with the Leica TCS 4D confocal laser scanning microscopy system (Leica, Wetzlar, Germany). Each slide was evaluated by two separate investigators in a blinded manner.

**References**

1. Li HY, Chien Y, Chen YJ, Chen SF, Chang YL, et al. (2011) Reprogramming induced pluripotent stem cells in the absence of c-myc for differentiation into hepatocyte-like cells. Biomaterials 32: 5994-6005.
2. Kao CL, Tai LK, Chiou SH, Chen YJ, Lee KH, et al. (2010) Resveratrol promotes osteogenic differentiation and protects against dexamethasone damage in murine induced pluripotent stem cells. Stem Cells Dev 19: 247e58.
3. Chen SJ, Chang CM, Tsai SK, Chang YL, Chou SJ, et al. (2010) Functional improvement of focal cerebral ischemia injury by subdural transplantation of induced pluripotent stem cells with fibrin glue. Stem Cells Dev 19: 1757-1767.
4. [Mou H](http://www.ncbi.nlm.nih.gov/pubmed?term=Mou H%5BAuthor%5D&cauthor=true&cauthor_uid=22482504), [Zhao R](http://www.ncbi.nlm.nih.gov/pubmed?term=Zhao R%5BAuthor%5D&cauthor=true&cauthor_uid=22482504), [Sherwood R](http://www.ncbi.nlm.nih.gov/pubmed?term=Sherwood R%5BAuthor%5D&cauthor=true&cauthor_uid=22482504), [Ahfeldt T](http://www.ncbi.nlm.nih.gov/pubmed?term=Ahfeldt T%5BAuthor%5D&cauthor=true&cauthor_uid=22482504), [Lapey A](http://www.ncbi.nlm.nih.gov/pubmed?term=Lapey A%5BAuthor%5D&cauthor=true&cauthor_uid=22482504), et al. (2012) Generation of multipotent lung and airway progenitors from mouse ESCs and patient-specific cystic fibrosis iPSCs. [Cell Stem Cell](http://www.ncbi.nlm.nih.gov/pubmed/22482504) 10: 385-397.
5. [Kadzik RS](http://www.ncbi.nlm.nih.gov/pubmed?term=Kadzik RS%5BAuthor%5D&cauthor=true&cauthor_uid=22482501), [Morrisey EE](http://www.ncbi.nlm.nih.gov/pubmed?term=Morrisey EE%5BAuthor%5D&cauthor=true&cauthor_uid=22482501) (2012) Directing lung endoderm differentiation in pluripotent stem cells. [Cell Stem Cell](http://www.ncbi.nlm.nih.gov/pubmed/22482501) 10: 355-361.

**Figure legends**

**Figure S1. High tidal volume ventilation increased iPSCs trafficked in the lung.** Representative photomicrographs (x400) with Hoechst (blue) immunofluorescent staining of frozen lung sections were from (A) control, non-ventilated mice and (B) mice ventilated at V_T_ 30 ml/kg for 4 h with room air. (C) The scattered density of the incorporated iPSCs in the lung was quantified as an average number of Hoechst-labeled iPSCs in 10 nonoverlapping fields of lung sections. Positive blue staining in the lung epithelium and interstitium is identified by arrows. The positive staining of Hoechst in the lung sections of mice increased after mechanical ventilation at V_T_ 30 ml/kg for 4 h compared with that of control, nonventilated mice. Data shown here are the mean ± SD of four independent experiments. *P< 0.05 vs. Non-ventilated control treated with PBS. Scale bars represent 20 μm. iPSCs= induced pluripotent stem cells; PBS = phosphate-buffered saline.

**Figure S2.** **LPS-induced ALI in mice treated by iPSCs/iPSC-CM and MEF/MEF-CM.** We used the intratracheal injection of LPS in C57BL/6 mice to induce acute lung injury. To investigate the treatment effect of iPSCs and iPSC-derived conditioned medium, we further injected the iPSCs, MEF, iPSC-CM, and MEF-CM into the mice of LPS-induced ALI through tail vein. Our results showed that both iPSCs and iPSC-CM significantly improved the lung injury in LPS-induced ALI in mice as compared to those of MEF or MEF-CM-treated mice. Importantly, the results of microarray analysis showed that both iPSCs and iPSC-CM could modulate the similar gene cluster expression in the lung lesions of LPS-induced ALI mice, suggesting that there existed the common trait of the biomolecular signatures in response to LPS-induced lung injury between the iPSCs and conditioned medium of iPSCs. ALI= acute lung injury; iPSC-CM = the conditioned medium of iPSCs; LPS= lipopolysaccharide; MEF= mouse embryonic fibroblasts; MEF-CM: the conditioned medium of MEF.

**Figure S3. iPSCs or iPSC-CM dose-dependently attenuated high-tidal-volume-induced lung injury and neutrophil infiltration.** The effects of administering iPSCs or iPSC-CM on (A) the quantification of airway structural damage and (B) neutrophil infiltration in bronchoalveolar lavage fluid in wild-type mice receiving mechanical ventilation at a high tidal volume (V_T_30) are shown. Data shown here are the mean ± SD of four independent experiments. *P< 0.05 vs. V_T_30-ventilated mice treated with PBS.
